# Factors Influencing Nurses’ Work–Life Balance: A Systematic Review of Challenges, Opportunities and Strategic Solutions for Healthcare Practice and Policy

**DOI:** 10.1155/jonm/5981858

**Published:** 2026-05-19

**Authors:** Ningshan Yang, Yu Xuan Ng, Chen Xing Yeoh Lui, Brigitte Fong Yeong Woo, Sok Ying Liaw

**Affiliations:** ^1^ Alice Lee Centre for Nursing Studies, Yong Loo Lin School of Medicine, National University of Singapore, Singapore, nus.edu.sg; ^2^ Department of Nursing, Alexandra Hospital, Singapore, ah.com.sg; ^3^ Nursing Administration, Ng Teng Fong General Hospital, Singapore

**Keywords:** job control, job demand, nurses, shift work, systematic review, work–life balance

## Abstract

**Aim:**

To consolidate existing evidence on the factors affecting nurses’ work–life balance.

**Background:**

Work–life balance is an emerging concept among many professions, including nursing. Given the global nursing shortage and high demand for nurses, more attention should be placed on understanding the factors affecting nurses’ work–life balance. Better measures can hence be created to improve nurses’ work–life balance.

**Methods:**

Six databases (PubMed, Excerpta Medica Database, Cumulative Index to Nursing and Allied Health Literature, PsycINFO and Scopus and Web of Science) were searched from inception up to February 2024. A grey literature search and screening of reference lists of relevant papers were also conducted. Identified studies were screened against the eligibility criteria, including population, outcome and type of study. The screening, appraisal and data extraction of the eligible papers were done independently by two reviewers. Available evidence was then synthesised using a convergent integrated approach.

**Results:**

Twenty‐one quantitative studies and one qualitative study were included. Six themes on factors affecting nurses’ work–life balance were derived: (1) sociodemographic characteristics, such as age, gender, income status and marital status, (2) personal life, including family support and social life, (3) job control, with emphasis on control over working hours, (4) job demand, encompassing both physical and psychological impacts, (5) work schedule, with highlight on frequent night shifts and shift changes and (6) working environment, including work culture and settings.

**Discussion:**

As evident from this review, work–life balance is a multifactorial concept. Nurses, nurse leaders and healthcare organisations have the shared responsibility of ensuring that nurses have an improved work–life balance. Understanding the different factors affecting nurses’ work–life balance can allow these stakeholders to take steps towards improving nurses’ work–life balance, which in turn result in better clinical outcomes. However, it should be considered that the evidence gathered in this study might not provide a complete overview of the factors affecting nurses’ work–life balance due to the complexities of the topic.

**Conclusion and Implications for Nursing and/or Health Policy:**

This review provides insight into the factors affecting the work–life balance of nurses and emphasises the need for the involved stakeholders to create strategies including safe‐care interventions, flexible working hours and family‐friendly policies to enhance nurses’ work–life balance.

## 1. Introduction

Changes in gender roles, careers and families are causing work–life balance to receive greater attention recently [1]. Younger generations increasingly value work–life balance, and they expect it to be included in their employment [2–4]. Nonetheless, work–life balance is not a theoretically developed concept despite being exhaustively studied, with varying definitions within the existing literature [5–7].

There are two commonly cited definitions of work–life balance. First, a definition proposed by Kalliath & Brough [8] states that work–life balance is the subjective perception that harmony exists between one’s personal and professional life, hence fostering personal development in alignment with one’s current life priorities. Second, maintaining proper equilibrium between one’s job and private life is the other suggested definition of work–life balance [9, 10].

Despite differing definitions of work–life balance, two key theories that have been studied are Border Theory [11] and Boundary Theory [12]. Both theories discuss work and life as domains separated by boundaries. These boundaries allow integration and segmentation of the domains depending on factors such as an individual’s preferences and careers [13]. For integration, both domains are recognised as the same, while segmentation occurs when the domains are mutually exclusive [14]. Individuals who can attain better integration between the two domains have better work–life balance [15]. This examination of boundaries can help us to comprehend the level of control that one has over their work–life balance [16]. However, even with its long history, theories revolving around work–life balance are still ever‐evolving due to social, economic, political and organisational influences [17].

Nursing is a stressful and demanding profession which poses great challenges towards managing boundaries between work and life domains [18]. Irregular working hours, work overload and occupational hazards cause imbalances between nurses’ work and lives [19, 20], hindering them from achieving work–life balance [21]. Moreover, apart from providing patients with professional care during their shifts, many nurses assume caregiver roles in their own families [22]. This further blurs the line between nurses’ careers and personal lives, hence impeding work–life balance.

At an individual level, poor work–life balance can result in many negative outcomes such as stress, health problems, job dissatisfaction, poor job performance and burnout [23–25]. At an organisational level, such ill‐effects are compounded rapidly resulting in workplace violence, medical errors, reduced productivity, poor quality of care and high attrition rates [19, 26, 27]. As a result, poor outcomes will be observed in both the individual and organisational level, giving rise to higher healthcare costs, poor patient satisfaction and nursing shortage [26, 27]. However, having good work–life balance can create many positive outcomes as well. Achieving a good work–life balance can improve nurses’ health and life satisfaction. It can also reduce stress and burnout and improve retention rates [24, 28]. Nurses’ work performance and quality of care provided will also improve with better work–life balance, which in turn enhances patient safety and satisfaction [29, 30].

An initial search of the Cochrane Database of Systematic Reviews, Joanna Briggs Institute (JBI) Database of Systematic Reviews and Implementation Reports and The International Prospective Register of Systematic Reviews (PROSPERO) revealed a paucity of secondary studies specifically examining the factors influencing nurses’ work–life balance. Two related scoping reviews were found, with one exploring the work–life balance among nursing faculty [31] and another studying work–life balance measurement tools within the healthcare sector [7]. However, both of these studies are not our areas of focus. A systematic review titled ‘A comprehensive review of the factors affecting the work‐life balance of nurses’ was found [32]. However, the review lacked components of an academic research paper, namely, the methods, results and discussion portions [33]. After extensive searching, no other secondary studies were found to study factors affecting nurses’ work–life balance. Hence, this systematic review aims to consolidate existing evidence on factors affecting nurses’ work–life balance. This review questions what are the factors affecting the work–life balance of nurses and their implications on nurses’ work–life balance.

### 1.1. Design

This mixed‐methods systematic review conducted according to the Preferred Reporting Items for Systematic Reviews and Meta‐Analyses (PRISMA) guidelines [34] (Appendix A) and the PRISMA 2020 for abstracts [34] (Appendix B) had the purpose of collating both quantitative and qualitative evidence to create a broader and deeper understanding of the research question [35]. It followed the JBI methodology using a convergent integrative review design [36]. A protocol was registered on PROSPERO (Registration number: CRD42024493150).

## 2. Materials and Methods

### 2.1. Search Strategy

A preliminary search was conducted, and keywords were analysed. A comprehensive search strategy was created by following the Peer Review of Electronic Search Strategies 2015 Evidence‐Based‐Checklist [37] (Appendix C). Synonyms related to ‘work–life balance’ and ‘nurses’ were included in different permutations and combinations and combined with the usage of the Boolean operators ‘AND’ and ‘OR’ (Supporting Table 1). The search strategy was confirmed with the help of two senior researchers (Yu Xuan Ng, Sok Ying Liaw) and searched in the following six electronic databases: PubMed, Excerpta Medica Database (EMBASE), Cumulative Index to Nursing and Allied Health Literature (CINAHL), PsycINFO, Scopus and Web of Science. No limits or filters were used to reduce the possibility of excluding potential studies. OpenGrey and ProQuest were also searched to identify articles not included in the seven databases. Manual screening of reference lists of related articles was also conducted.

### 2.2. Eligibility Criteria

This review included studies involving all types of full‐time and part‐time nurses working in all clinical settings. Studies that focused on factors affecting nurses’ work–life balance were considered. Quantitative, qualitative and mixed‐method study designs were included. Peer‐reviewed journal articles are published in academic journals that are indexed in recognised bibliographic databases. This criterion ensured that all included studies met minimum standards for scholarly quality and dissemination. All articles from inception till February 2024 were considered, and only articles available in English were included.

Excluded from this review are studies that involved nursing faculty and prelicensure nursing students. Studies containing populations apart from nurses were excluded unless nurses constituted most of the participants (more than 70%). Studies on telehealth nursing were also not considered. Studies exploring interventions to improve nurses’ work–life balance were not considered. Secondary studies, theses, dissertations, editorials, commentaries, opinion articles and conference abstracts were excluded.

The eligibility criteria are presented by population, outcomes and the types of studies in Table 1.

**TABLE 1:** Study eligibility.

	Population	Outcomes	Types of studies
Inclusion	All types of full‐time and part‐time nurses working in all clinical settings	Factors affecting nurses’ work–life balance	Primary studies Quantitative, qualitative and mixed‐method study designs Peer‐reviewed scholarly articles published in academic journals that are indexed in recognised bibliographic databases. From inception till February 2024 Available in English
Exclusion	Nursing faculty and prelicensure nursing students Telehealth nursing Populations apart from nurses unless nurses constituted most of the participants (more than 70%)	Interventions to improve nurses’ work–life balance	Secondary studies Theses, dissertations, editorials, commentaries, opinion articles and conference abstracts

### 2.3. Selection Process

The article selection followed a four‐step process according to the PRISMA guidelines [34]. In the first step following the initial search, identified studies were uploaded into EndNote 20 [38], where the removal of duplicated studies occurred. The eligibility criteria were pilot tested on a sample of studies before the actual screening of articles to refine the criteria. Secondly, the studies’ titles and abstracts were reviewed against the refined criteria. For the third step, full texts of studies that met the inclusion criteria were retrieved and reviewed against the eligibility criteria again. Studies that failed to meet the inclusion criteria were excluded in the final step. This selection process was conducted independently by two reviewers (Ningshan Yang and Chen Xing Yeoh Lui), and disagreements were solved through consensus building with a third reviewer (Yu Xuan Ng).

### 2.4. Assessment of Methodological Quality

The Mixed‐Methods Appraisal Tool version 2018 [39] (Appendix D), which is used to appraise the methodological quality of quantitative, qualitative and mixed‐methods studies, was used to appraise all the primary studies. The number of ‘yes’ answers determined the scores of each study, and the studies were graded as ‘high’ (scores above 75%), ‘moderate’ (scores between 50% and 75%) or ‘low’ (scores below 50%) quality. No studies were excluded irrespective of their quality to ensure that this review was as comprehensive as possible [40]. All studies were assessed by two reviewers independently (Ningshan Yang and Chen Xing Yeoh Lui). Inter‐rater reliability test was conducted to check for the level of agreement between the two reviewers, and a kappa statistic of 0.90 was attained. A third reviewer (Yu Xuan Ng) was approached to solve discrepancies.

### 2.5. Data Extraction

A customised data extraction checklist (Appendix E) was utilised to obtain the required information. The following components were included: the article title, author(s), year of publication, country where the study took place, study aim, study design, sample size and characteristics and key findings significant to this review. The checklist was pilot‐tested on a sample of included studies to ensure that the necessary data could be extracted [41]. Extracted data were then collated in an Excel spreadsheet. Missing data were ignored, and only available data were analysed. Data extraction was done independently by two reviewers (Ningshan Yang and Chen Xing Yeoh Lui), and discrepancies were solved through the intervention of a third reviewer (Yu Xuan Ng).

### 2.6. Data Analysis and Synthesis

A convergent integrated approach was adopted, where qualitative and quantitative data were merged and modified. A qualitative synthesis approach was then used to create analytical themes. Following data extraction, quantitative data were ‘qualitised’ through narrative interpretation of the data [42]. These data were translated into textual descriptions, allowing for integration with qualitative data. Themes were then identified after a detailed examination of the data. A three‐step thematic data synthesis was utilised for this review [43]. The data were first coded line‐by‐line before the codes were organised into categories in the second step, forming descriptive themes. The first two steps were conducted by the first reviewer (Ningshan Yang) before the second reviewer (Chen Xing Yeoh Lui). Checked through the categories. Lastly, the descriptive themes were compared with textual data, developing analytical themes. This step was done through discussions between the two reviewers (Ningshan Yang and Chen Xing Yeoh Lui). Finalised themes were presented thematically.

## 3. Results

### 3.1. Search Outcomes

**FIGURE 1:**
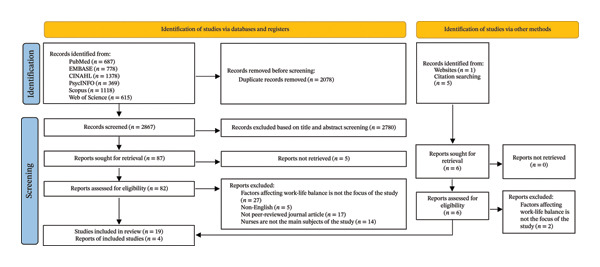
PRISMA flow diagram [34].

A total of 4747 studies were identified from database searches, where 2063 duplicates were removed. The titles and abstracts of 2684 articles were reviewed, and 82 studies were retrieved for full‐text screening after exclusion. Additionally, six articles were identified through manual screening of reference lists of related articles. Full‐text screening was also done for these articles. A total of 23 studies were included in this review. A PRISMA flow diagram (Figure 1) details the study selection process [34].

### 3.2. Study Characteristics

**FIGURE 2:**
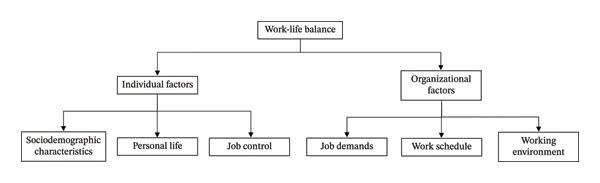
Framework for factors affecting work–life balance in nursing studies.

Of the 23 included studies, 22 were quantitative studies and there was one qualitative study. After the quality appraisal, 17 studies were rated high quality, five of moderate quality and one of low quality (Appendix F, Appendix G and Appendix H). The studies were conducted across various countries and multiple healthcare settings, involving different types of nurses. Fourteen studies were conducted in Asia, four in European countries, two in the United States of America (USA) and two in the Middle East. Healthcare settings included inpatient, outpatient, public, private, non‐governmental organisation and teaching hospitals, nursing homes, medical centres and community health centres. The sample size of the studies ranged from 60 to 7077 for quantitative studies and 26 for the qualitative study. Types of nurses involved in the studies include registered nurses, enrolled nurses and advanced practice nurses. The nurses were working either full‐time or part‐time. All quantitative studies used surveys and questionnaires as their data collection method while the qualitative study used semi‐structured interviews to collect the data. The study characteristics and summaries of key findings are detailed in Table 2. Extracted data were categorised into six themes (Figure 2): (1) sociodemographic characteristics, (2) personal life, (3) job control, (4) job demands, (5) work schedule and (6) working environment.

**TABLE 2:** Characteristics and findings of included studies.

Author (year), country	Study aim	Study design	Sample size (*n*) and characteristics	Data collection and outcome measurements	Key findings (themes)	Quality of paper
Cohen and Kirchmeyer (2005) [44]; Israel	To examine the differential effects of both work and nonwork demands and inter‐role management on work/nonwork conflict, turnover intention, turnover and absenteeism across three ethnic groups.	Quantitative: Survey	*n* = 233 Female nurses from 2 hospitals with mostly Arab nurses and 1 hospital with predominantly Jewish nurses	Questionnaires Shamir’s [45] 6‐item scale of interdomain conflict	Ethnicity (sociodemographic characteristics) Job status, job tension (job demands)	High

Arasi and Sathish (2005) [46]; India	To evaluate the work–life balance among nursing professional during COVID‐19 s wave.	Quantitative: Descriptive cross‐sectional survey	*n* = 89 Nurses from central government hospitals, private hospitals, primary health centres, community health centres and a non‐governmental organisation hospital	Survey Tool that was structured and validated under 7 experts in the field of nursing	Patient load (job demands) Working hours, shiftwork (work schedule)	Moderate

Lee et al. (2020) [24]; Taiwan	To use a well‐developed work–life climate scale to measure the nurses’ work–life balance from their behaviours rather than their perceptions as well as observe how their behaviours change from time to time.	Quantitative: Retrospective	*n* = 2016 Nurses from a regional teaching hospital	Internal data sets of 2014–2018 based on nurses from the work–life balance dimension of the Chinese version of the Safety Attitudes Questionnaire (SAQ) Work–life balance dimension of the Chinese version of the SAQ borrowed from the work–life climate developed by Sexton et al. [47]	Experience in organisation, experience in position (sociodemographic characteristics) Reporting events in the past 12 months (job demands)	High

Shiffer et al. (2018) [48]; Italy	To investigate if the direction of rapid shift rotation schedules affects hospital nurses’ sleep quantity and quality, work performance and its impact on their social and family life.	Quantitative: Cross‐sectional	*n* = 100 Registered female nurses from two major hospitals	Closed question questionnaire and daily diary	Shift rotation schedules (work schedule)	High

Dyrbye et al. (2020) [49]; United States of America	To determine the incidence of burnout, explore personal and professional factors independently associated with burnout and satisfaction with work–life integration, and compare the prevalence of burnout and satisfaction with work‐life integration among advance practice nurses with other United States workers.	Quantitative: Survey	*n* = 976 Advanced practice nurses from hospital, outpatient and other healthcare settings	Survey Indication of agreement with the statement: ‘My work schedule leaves enough time for my personal/family life’ [50, 51]	Marital status, having children (sociodemographic characteristics) Working hours (work schedule)	High

Dyrbye et al. (2019) [52]; United States of America	To evaluate the current state of burnout and satisfaction with work‐life integration among United States nurses, explore the personal and professional characteristics associated with burnout and satisfaction with work‐life integration, and compare the prevalence of burnout and satisfaction with work–life integration among nurses to United States workers in other fields.	Quantitative: Survey	*n* = 7077 Nurses who were members of the American Nurses Association	Survey Indication of agreement with the statement: ‘My work schedule leaves enough time for my personal/family life’ [50, 51]	Experience in position (sociodemographic characteristics) Working hours (work schedule)	High

Ng et al. (2017) [53]; Taiwan	To examine the extent to which the job demands and job control of nurses are related to their work–life balance.	Quantitative: Survey	*n* = 2040 Registered nurses from eight private hospitals, covering one medical centre and seven community teaching hospitals	Questionnaire Work–life balance measurement was adapted from Hayman [54]	Job demand (job demands) Job control (job control)	High

Karunagaran et al. (2020) [55]; India	To evaluate the work–life balance of nurses during the pandemic.	Quantitative: Descriptive	*n* = 220 Nurses from a tertiary hospital	Survey Work–life Balance Scale developed by Fisher [56] and later modified by Hayman [54]	Religion (sociodemographic characteristics)	High

Pryce et al. (2006) [57]; Denmark	To assess the impact of an open‐rota scheduling system on health, job satisfaction and work–life balance and to evaluate the processes involved in the uptake of the intervention, the maintenance of the intervention in order to guide the implementation of open‐rota systems.	Quantitative: Randomised controlled trial	*n* = 177 Nurses and healthcare workers working together in multi‐disciplinary teams from a psychiatric hospital in Denmark	Questionnaire survey Five single‐item measures of work–life balance developed for the study	Open‐rota systems (job control)	Moderate

Korkmaz Aslan et al. (2023) [58]; Turkey	To determine the factors affecting the work–life balance and psychological endurance levels of nurses working in internal clinics.	Quantitative: Descriptive and relational	*n* = 472 Nurses working in various hospital and internal units	Survey Work–life balance scale developed by Apaydın [59]	Marital status, having children, educational status, income status (sociodemographic characteristics) Working hours, night shifts, working at unusual times, flexible overtime (work schedule)	Moderate

Okayasu et al. (2022) [60]; Japan	To investigate the relationship between nurses and work–self balance at different phases in life, such as age, marriage and raising children, and the occupational factors that influence work–self balance.	Quantitative: Cross‐sectional	*n* = 1063 Nurses working at Dokkyo Medical University Hospital	Survey New Brief Job Stress Questionnaire (New BJSQ) [61]	Age, marital status, having children (sociodemographic characteristics)	High

Navajas‐Romero et al. (2020) [62]; European countries	To analyse how factors lead to a greater or lesser degree of work–life balance, by adopting the Job demand–control–support model.	Quantitative: Retrospective	*n* = 991 Nursing professionals	European Working Conditions Survey (EWCS) Indicator composed of 4 questions	Physical demands, psychological demands (job demands)	High

Min (2022) [63]; South Korea	To explore the effect of resilience burnout, and work‐related physical pain on the work–life balance of RNs.	Quantitative: Cross‐sectional	*n* = 155 Registered nurses from 37 nursing homes	Questionnaire Korean version of the work–life balance tool [64]	Resilience (personal life) Work‐related physical pain, burnout (job demands)	High

Gribben and Semple (2021) [65]; Ireland	To examine the prevalence and predictors of burnout and work–life balance among the haematology cancer nursing workforce in Ireland.	Quantitative: Non‐experimental, cross‐sectional survey	*n* = 78 Registered haematological nurses working in inpatient and outpatient settings	Survey Modification of the single‐item scale used by Shanafelt and colleagues [50, 51, 66] and [67], and four other Likert‐style questions developed by the research team, based on literature and expert opinion	Having children (sociodemographic characteristics) Clinical setting (working environment)	High

Jena et al. (2021) [68]; India	To determine the relationship between emotional reflexivity and work–life integration through the mechanism of moral courage and enhance our understanding of the importance of these nursing concepts to enable the nurses to develop better coping strategies for work–life integration.	Quantitative: Cross‐sectional	*n* = 249 Nurses employed in 17 reputed public and private hospitals	Questionnaire 16‐item Work–Life Boundary Enactment scale developed by Wepfer and colleagues [69]	Emotional reflexivity, courage (personal life)	High

Watanabe and Yamauchi (2016) [70]; Japan	To examine the direct and indirect effects of psychological factors of overtime work on employee well‐being: involuntary and voluntary overtime work on work–nonwork balance satisfaction at both individual and workplace levels using a multilevel structural equation modelling (SEM) technique in the hierarchal structured sample of nurses nested in workplaces.	Quantitative: Survey	*n* = 603 Full‐time working nurses (excluding enrolled nurses) from three hospitals	Survey	Overtime work (work schedule)	Moderate

Adiba and Nair (2021) [71]; India	To find out the factors of occupational stress and its impact on work and work life balance of nursing professionals in a large private hospital in south India.	Quantitative: Descriptive	*n* = 60 Permanent nurses in a large private hospital	Questionnaire Work–life balance variables	Job stress (job demands) Working hours, shiftwork, overtime work, meetings/training after office hours (work schedule)	Low

Oh & Cho (2020) [72]; South Korea	To identify the factors affecting work–life conflict of nurses.	Quantitative: Survey	*n* = 271 Shift nurses from four secondary care hospitals	Survey Scale developed by Carlson, Kacmar and Williams [73]	Leisure constraints (personal life) Control over specific start and finish times of work (job control) Frequency of swapping shifts with colleagues (work schedule)	High

Rony and Alamgir (2023) [21]; Bangladesh	To investigate the relationship between work–life imbalance and job dissatisfaction, and the impact of work on family life and vice versa.	Quantitative: Cross‐sectional	*n* = 656 Nurses from various healthcare settings	Questionnaire 15 Likert‐scale questions developed by adapting and modifying existing literature	Age, gender, income status, experience (sociodemographic characteristics) Working hours (work schedule) Employer type, working department (working environment)	High

Dousin et al. (2021) [74]; Malaysia	To explore how a specific context supports or hinders work–life balance experiences focusing on women doctors and nurses in Malaysia.	Qualitative: Interpretivist inquiry paradigm	*n* = 26 Full‐time doctors and nurses from three public and three private hospitals	Semi‐structured interviews	Gender (sociodemographic characteristics) Staff shortage (job demands) Collectivist work culture (working environment)	Moderate

Sripo et al. (2019) [75]; Thailand	To explore the factors corelated with the work–life balance of occupational health nurses by applying the work–life balance concept of Greenhaus, Collins, & Shaw [76].	Quantitative: Cross‐sectional	*n* = 287 Occupational nurses working in every type of business facility	Questionnaire Questionnaire on work–life balance of Sayamon Akekulanan [77]; which was created based on the concept of work–life balance of Greenhaus, Collins & Shaw	Age, marital status, income sufficiency, experience in organisation (sociodemographic characteristics) Family support (personal life) Role ambiguity, role conflict (job demands) Working hours (work schedule) Workplace support (working environment)	High

Nurumal et al. (2017) [78]; Malaysia	To examine work–life balance and its related factors among teaching hospital nurses. To identify factors of social demography, job nature and quality of life towards work–life balance among nurses.	Quantitative: Cross‐sectional	*n* = 1002 Nurses from a teaching hospital	Surve Adapted NIOSH Generic Job Stress Questionnaire [79], Sense of Coherence questionnaires from Antonousky [80] and Quality of Life questionnaires from WHO [81]	Non‐work activities, social life, quality of life: physical, psychological, social, environmental (personal life) Task control, decision control, physical environment control. resource control, management (job control) Job requirement (job demands) Fixed shift, annual leaves (work schedule) Supervisor support, environmental support (working environment)	Moderate

Terzi & Azizoğlu (2023) [82]; Turkey	To investigate the relationship between intensive care nurses’ workload perception and work–life balance during the COVID‐19 pandemic.	Quantitative: Cross‐sectional, descriptive and correlational	*N* = 925 Intensive care nurses who were members of the Turkish Intensive Care Nurses Association	Survey 5‐point Likert‐scale developed by Apaydin	Workload (job demands) Shift length (work schedule)	High

### 3.3. Data Synthesis

#### 3.3.1. Sociodemographic Characteristics

Eleven studies explored the impact of sociodemographic characteristics on nurses’ work–life balance. Regarding age, two studies reported that older nurses had better work–life balance [21, 75], while Okayasu et al. [60] examined age as a mediating factor in nurses’ work–life balance. When exploring gender, two studies done in Asia found that female nurses had poorer work–life balance than male nurses [21, 74]. Educational status was reported by Korkmaz Aslan et al. [58] to be significantly associated with nurses’ work–life balance. Nurses’ work–life balance also varied by income status, as explored by two studies [58, 75], and Rony et al. [21] specified that low salaries hindered nurses’ work–life balance. Regarding experience, three studies found that working experience improved nurses’ work–life balance [21, 24, 52]. However, while Sripo et al. [75] noted that more experience in the organisation positively affected nurses’ work–life balance, Lee et al. [24] found no definite pattern between them. Ethnicity was explored by Cohen & Kirchmeyer [44] as a mediating factor of nurses’ work–life balance, while Karunagaran et al. [55] reported that being spiritual improved nurses’ work–life balance.

For marital status, Korkmaz Aslan et al. [58] noted it to be significantly associated with nurses’ work–life balance. Two studies reported that single nurses had better work–life balance [60, 75]. However, Dyrbye et al.’s [52] study conducted in the United States of America (USA) found that marriage improved nurses’ work–life balance. Similarly, when exploring having children, while three studies reported it to hinder nurses’ work–life balance [58, 60, 65], Dyrbye et al. [49] found the opposite to be true.

#### 3.3.2. Personal Life

Personal life factors were explored in five studies. One study reported that family support improved nurses’ work–life balance [75]. Oh & Cho [72] found that leisure constraints hindered nurses’ work–life balance. In Nurumal et al.’s [78] study, better physical, psychological, social and environmental QoL was reported to improve nurses’ work–life balance, while non‐work activities were reported to have a negative relationship with nurses’ work–life balance. Social life was also noted to be significantly associated with nurses’ work–life balance in Nurumal et al.’s [78] study. Resilience [63], courage and emotional reflexivity [68] were noted to improve nurses’ work–life balance.

#### 3.3.3. Job Control

Job control was examined by four studies. Ng et al. [53] reported inconclusive results on the impacts of job control on nurses’ work–life balance, while Nurumal et al. [78] noted that job control hindered nurses’ work–life balance. Two studies reported that control over working hours improved nurses’ work–life balance [57, 72]. One study was a randomised controlled trial, which trialled a system whereby nurses planned their individual work schedules. The intervention group reported better work–life balance, indicating that having more control over working hours improved nurses’ work–life balance.

#### 3.3.4. Job Demands

Job demands were highlighted across 11 studies. Two studies found that job demands hindered nurses’ work–life balance [53, 62]. However, while Ng et al. [53] reported greater negative impacts from psychological demands than physical demands, Navajas‐Romero et al. [62] noted otherwise. Three studies reported that high workload hindered nurses’ work–life balance [46, 74, 82], with Dousin et al. [74] and Arasi & Sathish [46] exploring staff shortage and excessive patient load as predictors of high workload, respectively. Two studies explored job stress [44, 71], and one study explored high job requirements as barriers to nurses’ work–life balance [78].

Role conflict and role ambiguity were explored by Sripo et al. [75] to hinder nurses’ work–life balance. Cohen & Kirchmeyer’s [44] study reported that nurses working full time had poorer work–life balance. Min [63] explored work‐related physical pain and burnout as barriers to nurses’ work–life balance. Lee et al. [24] also found that reporting few events resulted in better work–life balance for nurses than not reporting any events.

#### 3.3.5. Work Schedule

Work schedule was explored in 12 studies. Two studies noted that shiftwork hindered nurses’ work–life balance [46, 71]. More night shifts, usual working times [58] and frequent shift changes [72] were also found to hinder nurses’ work–life balance. However, Nurumal et al. [78] found that nurses with fixed shifts reported better work–life balance than nurses with multiple or rotational shifts. Shiffer et al. [48] also reported that clockwise shift rotation improved nurses’ work–life balance compared to counterclockwise shift rotation.

Eight studies agreed that longer working hours resulted in poorer work–life balance for nurses. Nurumal et al.’s [78] study also noted that more annual leaves improved nurses’ work–life balance. Non‐flexible overtime [58, 71], having meetings and training after working hours [71] and involuntary overtime [70] were found to hinder nurses’ work–life balance. However, Watanabe & Yamauchi [70] noted that nurses’ work–life balance improved with voluntary overtime due to extrinsic motivations such as monetary incentives and recognition.

#### 3.3.6. Working Environment

Five studies explored nurses’ working environment. Two studies found that workplace support enhanced nurses’ work–life balance [75, 78], while a qualitative study noted that a collectivist work culture improved nurses’ work–life balance [74]. Regarding clinical settings, Gribben & Semple [65] found that inpatient nurses had poorer work–life balance than outpatient nurses. Rony et al. [21] reported that government sector nurses had better work–life balance than private job‐holder nurses. General ward nurses were also noted to have better work–life balance than intensive care unit (ICU), operating theatre (OT) and emergency department (ED) nurses [21].

## 4. Discussion

Many sociodemographic characteristics associated with work–life balance were identified in this review. Age was identified as an independent and mediating factor in nurses’ work–life balance. This review and past studies identified older nurses to have better work–life balance due to their ability to draw better work–nonwork boundaries [83, 84]. Moreover, it was observed that the wisdom accrued with age plays a pivotal role in moderating the interplay between various factors and the attainment of work–life balance. This phenomenon is attributable to the evolving work demands and shifting perceptions of work–life equilibrium across different life stages [85].

This review also suggested that female nurses had poorer work–life balance compared to their male counterparts. Past studies have cited gender bias and cultural norms as barriers to achieving work–life balance for female employees [86]. This is especially true in Asia, where traditional views that women’s roles should be confined to the household remain [87]. Female nurses hence carry dual responsibilities of personal and professional duties, increasing the tension between work and life.

While this review highlights the positive impacts of income status on nurses’ work–life balance, previous studies yielded inconclusive findings regarding the relationship between income and work–life balance. This inconsistency can be attributed to the diverse definitions of income employed across different studies [88], leading to varying outcomes and lack of consensus.

In line with previous research, the review corroborates the role of ethnicity as a mediating factor in work–life balance [44], which can be attributed to inequalities faced by different ethnicities in the workforce [89], as well as differences in cultural values and religious beliefs, which can affect perceptions towards work–life balance [90]. However, past studies have also shown contradicting findings, whereby ethnicity was not a moderator of work–life balance. Such non‐conclusive findings can be a result of the complex characteristics involved in ethnicity, such as religion, nationality, culture and beliefs [91]. Narrowing down on the effects of religion on nurses’ work–life balance, this review highlighted its beneficial influences [55], which is supported by past studies emphasising the importance of religiosity in supporting work–life balance of employees [92, 93].

Nurses’ experience was an emerging factor in this review that has not been extensively explored in past studies. There was consensus between the findings that nurses with more experience tend to have greater satisfaction with work–life balance [21, 24, 52, 75]. However, it was noted that working experience had a stronger effect on nurses’ work–life balance than experience in the organisation.

The evidence presented in this review reveals inconsistent findings regarding the influence of marital status and having children on nurses’ work–life balance. Past studies have similarly suggested that these factors may not exert significant effects on work–life balance [94]. Marital status and parental responsibilities often entail increased demands and reduced personal time, potentially leading to conflicts and hindering efficient work [95]. Nonetheless, as this review has underscored, strong family support can empower individuals to navigate job demands and mitigate occupational stress [96]. Family support and assistance in childcare can serve as crucial resources for nurses in harmonising their personal and professional lives [95]. Thus, the diverse family dynamics and amount of children support received among nurses may account for the variability in observed impacts on work–life balance, thereby elucidating the disparities in research findings.

This review identified personal attributes such as resilience, courage and emotional reflexivity as contributors to nurses’ enhanced work–life balance, aligning with the findings of Brough et al. [5], who reported that psychological capital positively impacts work–life balance. Therefore, it might be advantageous to introduce interventions in the workplace aimed at fostering positive attributes, such as resilience training [97].

Regarding job control, which encompasses decision authority and skill discretion [98], this review yielded varying evidence. Notably, skill discretion was not thoroughly explored. Professional autonomy holds significant importance in nursing [99], denoting nurses’ capacity to apply their professional knowledge to patient care [100]. However, the cultivation of professional autonomy necessitates adequate clinical knowledge and decision‐making skills [101]. Therefore, the absence of focus on skill discretion may explain the inconclusive results concerning job control.

Furthermore, a higher degree of control over working hours was found to enhance nurses’ work–life balance. Consistently, prior studies have highlighted the benefits of flexible working hours and self‐rostering, enabling nurses to tailor their schedules to their workloads [102–104]. This flexibility facilitated the balancing of work and family responsibilities [105], thus improving overall work–life balance.

Job demands encompass the various aspects of one’s role that necessitate continuous effort, thereby incurring both psychological and physiological costs [106]. While consensus exists regarding the negative impacts of both physical and psychological job demands on nurses’ work–life balance, conflicting evidence arose concerning which aspect holds greater influence. Disparities in working environments and organisational structures may account for these divergent findings. As highlighted in this review, nurses contend with elevated workloads, fatigue, exposure to traumatic and stressful events and emotional strain [107, 108]. These demands exact a toll on their physical and mental well‐being, disrupting their daily routines and complicating the management of work and family commitments [109].

Shiftwork represents a distinctive feature of the nursing profession, recognised in this review as a significant impediment to nurses’ work–life balance. The nature of shiftwork entails prolonged and unpredictable working hours, encroaching upon time allocated to familial, social and recreational pursuits, thereby undermining overall work–life equilibrium [110]. Moreover, this review delineated strategies to mitigate the work–life conflict arising from shiftwork among nurses, such as adhering to fixed shifts and implementing a clockwise rotation schedule. Consequently, the impacts of shiftwork on nurses’ work–life balance are contingent upon the intricate interplay between scheduling practices, organisational dynamics and individual characteristics [110].

Additionally, protracted working hours and overtime are prevalent occurrences in the nursing profession [111]. Consistent with prior research [112], this review concurred that these factors detrimentally affect nurses’ work–life balance. However, it also unveiled a nuanced finding: voluntary overtime prompted by extrinsic motivations enhanced nurses’ work–life balance. This discovery aligns with the observations of Yu & Leka [113], who proposed that exercising control over overtime and receiving adequate compensation can incentivise voluntary overtime, ultimately fostering improved work–life balance.

Both this review and previous research have identified workplace support as a key determinant of nurses’ work–life balance [114, 115]. Workplace support encompasses the provision of resources and policies aimed at facilitating the harmonisation of work and nonwork responsibilities [96, 114]. Moreover, this review revealed that a collectivist work culture fosters collaboration among colleagues, thereby enhancing social support in the workplace [102, 116], ultimately contributing to the improvement of nurses’ work–life balance.

Nurses have the flexibility to choose from various clinical settings, and this review delves into how these choices impact their work–life balance. This review has highlighted that working in hospitals tends to correlate with poorer work–life balance compared to outpatient settings [117]. This can be attributed to workplace‐specific factors and variations in flexibility. Similarly, the review and older studies have indicated that nurses in the private‐sector typically enjoy more control over working hours, leading to better work–life balance compared to their counterparts in the government sector [118].

Furthermore, the review also identified that nurses in ICU, OT and ED generally experience poorer work–life balance than those in general ward settings. While previous studies have not extensively compared work–life balance of nurses across different departments, nurses in ICU, OT and ED have consistently reported challenges due to stressful work environments and high workloads [119–121]. As abovementioned, variations in working environments across settings lead to differing job demands, thus impacting nurses’ work–life balance in distinct ways.

### 4.1. Strengths and Limitations

This comprehensive review incorporated studies encompassing diverse nursing roles across various clinical settings and included research from multiple countries, providing valuable insight into the work–life balance of nurses across different cultural and societal contexts.

However, there are several limitations to this review, which should be acknowledged. Firstly, the included studies used varying data collection methods and outcome measures, possibly leading to discrepancies and inconsistencies in the findings. Another limitation would be the lack of details on the direction in which specific factors affect nurses’ work–life balance in the included articles, hence limiting the depth of analysis possible in this review. It is also important to note that this review did not consider articles published in non‐English languages and grey literature, which leaves open the possibility that some relevant literature may have been overlooked. Additionally, inconsistencies in the use of terminology may have contributed to the omission of certain studies during the screening process.

### 4.2. Direction for Future Research

In this review, it has been noted that there is a lack of a proper definition of work–life balance, which hindered the consistency of the findings. Hence, a universal concept should first be developed to ensure standardisation across studies.

The primary focus of this review was on individual and organisational factors influencing nurses’ work–life balance. However, future research should place greater emphasis on social and societal factors to gain a comprehensive understanding of the issue.

Additionally, the review has identified many inconclusive results, especially in multivariate models, underscoring the need for future studies to explore the complex interplay between the factors affecting nurses’ work–life balance. Investigating all dimensions of these factors could lead to more conclusive findings.

Furthermore, since this review is a consolidation of the factors affecting nurses’ work–life balance, future research can delve into specific factors to gain a deeper understanding of the individual factors affecting nurses work–life balance.

Lastly, a considerable proportion of the included studies used cross‐sectional and retrospective study designs. Hence, there were challenges in determining the causal relationships of the factors affecting work–life balance. As such, future studies can consider study designs such as randomised controlled trials or longitudinal studies to explore if such relationships are indeed true.

### 4.3. Implications for Nursing and Health Policy

Noting the significant impact of personal attributes on nurses’ work–life balance, organisations should implement initiatives to improve nurses’ psychological capital. Examples include stress management and resilience‐building workshops to equip nurses with coping and self‐care strategies. Moreover, the review emphasises the importance of implementing family‐friendly policies to support nurses in balancing their professional and personal responsibilities. Such responsibilities, as highlighted by Ko [122], can contribute significantly to improving nurses work–life balance.

This review also identified that varying working environments give rise to different job demands. It is therefore recommended that policymakers identify the more prominent aspects of job demand affecting the work–life balance of nurses within their specific organisational context to inform the development of targeted strategies aimed at mitigating these demands and improving overall nurses’ work–life balance. In terms of working hours, it was noted that control over work schedules and incentivised overtime work alleviated work–life conflicts faced by nurses. This emphasises the need for organisation to ensure adequate worker control over work schedule [123] and increase compensation for nurses working long hours and overtime. Given ample evidence on the benefits of self‐scheduling and flexible working hours, hospital administrators should implement such policies appropriately to maximise nurses’ work–life balance.

However, these policies are also faced with challenges, and their success depends on contextual factors and implementation processes [124]. The working environment and dynamics should be taken into consideration before the execution of such strategies. Nurse leaders will also need to strike a balance between staffing requirement and nurse’ needs to ensure successful implementation of such policies [125]. Further research should also be done on employees’ attitudes towards such policies, and their benefits and limitations, to ensure their proper implementation and utilisation.

## 5. Conclusion

This review thoroughly examined the multifaceted factors influencing nurses’ work–life balance, identifying six distinct themes categorised into individual and organisational factors. In achieving work–life balance, nurses, nurse leaders and healthcare organisations all play pivotal roles. The findings underscored various aspects that stakeholders can address to enhance nurses’ work–life balance, ultimately leading to improved outcomes at both the individual and organisational levels. Given the dynamic nature of work–life balance concepts and expectations, the factors impacting nurses’ work–life balance are expected to evolve over time. Therefore, research in this area should remain ongoing and adaptive. Future studies should delve deeper into the intricate relationships between various factors and nurses’ work–life balance, allowing for the development of targeted interventions tailored to the unique needs of individual organisations. By continuously exploring these factors, stakeholders can contribute to creating a supportive work environment that promotes nurses’ well‐being and enhances their ability to provide quality patient care.

## Author Contributions

Study conception/design: Ningshan Yang, Yu Xuan Ng and Sok Ying Liaw.

Data collection: Ningshan Yang and Chen Xing Yeoh Lui.

Data analysis: Ningshan Yang, Yu Xuan Ng and Chen Xing Yeoh Lui.

Study supervision: Sok Ying Liaw and Brigitte Fong Yeong Woo.

Manuscript writing: Ningshan Yang, Yu Xuan Ng and Brigitte Fong Yeong Woo.

Critical revisions for important intellectual content: Yu Xuan Ng, Sok Ying Liaw and Brigitte Fong Yeong Woo.

## Funding

This work was supported by the National University of Singapore Research Fellow Start‐up Grant.

## Conflicts of Interest

The authors declare no conflicts of interest.

## Supporting Information

Additional supporting information can be found online in the Supporting Information section.

## Supporting information


**Supporting Information** Supporting File 1: Detailed search strategy. Appendix A: Preferred Reporting Items for Systematic Reviews and Meta‐Analyses 2020 Checklist. Appendix B: Preferred Reporting Items for Systematic Reviews and Meta‐Analyses 2020 for Abstracts Checklist. Appendix C: Peer Review of Electronic Search Strategies 2015 Evidence‐Based Checklist. Appendix D: Mixed‐Methods Appraisal Tool version 2018. Appendix E: Customised data extraction checklist. Appendix F: Quality appraisal of quantitative descriptive studies using the Mixed‐Methods Appraisal Tool version 2018. Appendix G: Quality appraisal of quantitative randomised controlled trials using the Mixed‐Methods Appraisal Tool version 2018. Appendix H: Quality appraisal of qualitative studies using the Mixed‐Methods Appraisal Tool version 2018.

## Data Availability

The literature data supporting this scoping review are from previously reported studies and datasets, which have been cited. The processed data are available within the text.
