# Supplementary material for: Factors Influencing Nurses’ Work–Life Balance: A Systematic Review of Challenges, Opportunities and Strategic Solutions for Healthcare Practice and Policy
Source: J Nurs Manag. 2026 May 19;2026:5981858. doi: 10.1155/jonm/5981858 (PMC13189454; doi:10.1155/jonm/5981858)
Supplement: Supplementary file 1 — Supporting Information Supporting File 1: Detailed search strategy. Appendix A: Preferred Reporting Items for Systematic Reviews and Meta‐Analyses 2020 Checklist. Appendix B: Preferred Reporting Items for Systematic Reviews and Meta‐Analyses 2020 for Abstracts Checklist. Appendix C: Peer Review of Electronic Search Strategies 2015 Evidence‐Based Checklist. Appendix D: Mixed‐Methods Appraisal Tool version 2018. Appendix E: Customised data extraction checklist. Appendix F: Quality appraisal of quantitative descriptive studies using the Mixed‐Methods Appraisal Tool version 2018. Appendix G: Quality appraisal of quantitative randomised controlled trials using the Mixed‐Methods Appraisal Tool version 2018. Appendix H: Quality appraisal of qualitative studies using the Mixed‐Methods Appraisal Tool version 2018. [file JONM-2026-5981858-s001.docx]

**Supporting information**

**Supplementary Table 1: Detailed search strategy**

Database: PubMed

Date of Search: 04/04/2024

No. of Results: 687

Limits applied *(refer to PRISMA-S item 9)*: nil

Filters applied *(refer to PRISMA-S item 10)*: nil

| **S/N** | **Search terms** | **Number of results** |
| --- | --- | --- |
| 1 | "Nurses"[Mesh] | 100262 |
| 2 | "nurs*"[Title/Abstract] OR "enrolled nurs*"[Title/Abstract] OR "registered nurs*"[Title/Abstract] OR "Community nurs*"[Title/Abstract] OR "Acute nurs*"[Title/Abstract] OR "Clinic nurs*"[Title/Abstract] | 551220 |
| 3 | #1 OR #2 | 585558 |
| 4 | "Work-Life Balance"[Mesh] | 1088 |
| 5 | ("Worklife"[Title/Abstract] OR "work life"[Title/Abstract] OR "work life"[Title/Abstract] OR "Work-nonwork"[Title/Abstract] OR "work self"[Title/Abstract] OR "work self"[Title/Abstract]) AND ("Balance"[Title/Abstract] OR "Conflict"[Title/Abstract] OR "Equilibrium"[Title/Abstract] OR "relation*"[Title/Abstract] OR "integration"[Title/Abstract] OR "harmony"[Title/Abstract] OR "blend"[Title/Abstract]) | 3868 |
| 6 | #4 OR #5 | 4449 |
| 7 | #3 AND #6 | 687 |

("Nurses"[MeSH Terms] OR ("nurs*"[Title/Abstract] OR "enrolled nurs*"[Title/Abstract] OR "registered nurs*"[Title/Abstract] OR "community nurs*"[Title/Abstract] OR "acute nurs*"[Title/Abstract] OR "clinic nurs*"[Title/Abstract])) AND ("Work-Life Balance"[MeSH Terms] OR (("Worklife"[Title/Abstract] OR "work life"[Title/Abstract] OR "work life"[Title/Abstract] OR "Work-nonwork"[Title/Abstract] OR "work self"[Title/Abstract] OR "work self"[Title/Abstract]) AND ("Balance"[Title/Abstract] OR "Conflict"[Title/Abstract] OR "Equilibrium"[Title/Abstract] OR "relation*"[Title/Abstract] OR "integration"[Title/Abstract] OR "harmony"[Title/Abstract] OR "blend"[Title/Abstract])))

**Supplementary Table 1 (continued)**

Database: Embase.com

Date of Search: 04/04/2024

No. of Results: 778

Limits applied *(refer to PRISMA-S item 9)*: nil

Filters applied *(refer to PRISMA-S item 10)*: nil

| **S/N** | **Search terms** | **Number of results** |
| --- | --- | --- |
| 1 | 'nurse'/exp | 227412 |
| 2 | nurs*:ab,ti OR 'enrolled nurs*':ab,ti OR 'registered nurs*':ab,ti OR ‘community nurs*’:ab,ti OR ‘acute nurs*’:ab,ti OR ‘clinic nurs*’:ab,ti | 651915 |
| 3 | #1 OR #2 | 705345 |
| 4 | 'work-life balance'/exp | 3290 |
| 5 | (worklife:ab,ti OR 'work life':ab,ti OR 'work-life':ab,ti OR 'work-nonwork':ab,ti OR 'work self':ab,ti OR 'work-self':ab,ti) AND (balance:ab,ti OR conflict:ab,ti OR equilibrium:ab,ti OR relation*:ab,ti OR integration:ab,ti OR harmony:ab,ti OR blend:ab,ti) | 4399 |
| 6 | #4 OR #5 | 5869 |
| 7 | #3 AND #6 | 778 |

('nurse'/exp OR (nurs*:ab,ti OR 'enrolled nurs*':ab,ti OR 'registered nurs*':ab,ti OR ‘community nurs*’:ab,ti OR ‘acute nurs*’:ab,ti OR ‘clinic nurs*’:ab,ti)) AND (('work-life balance'/exp) OR ((worklife:ab,ti OR 'work life':ab,ti OR 'work-life':ab,ti OR 'work-nonwork':ab,ti OR 'work self':ab,ti OR 'work-self':ab,ti) AND (balance:ab,ti OR conflict:ab,ti OR equilibrium:ab,ti OR relation*:ab,ti OR integration:ab,ti OR harmony:ab,ti OR blend:ab,ti)))

**Supplementary Table 1 (continued)**

Database: CINAHL (EBSCO)

Date of Search: 04/04/2024

No. of Results: 1378

Limits applied *(refer to PRISMA-S item 9)*: nil

Filters applied *(refer to PRISMA-S item 10)*: nil

| **S/N** | **Search terms** | **Number of results** |
| --- | --- | --- |
| 1 | (MH "Nurses+") | 238951 |
| 2 | TI ( Nurs* OR “Enrolled nurs*” OR “Registered nurs*” OR “Community nurs*” OR “Acute nurs*” OR “Clinic nurs*” ) OR AB ( Nurs* OR “Enrolled nurs*” OR “Registered nurs*” OR “Community nurs*” OR “Acute nurs*” OR “Clinic nurs*” ) | 590834 |
| 3 | #1 OR #2 | 681612 |
| 4 | (MH "Work-Life Balance") | 3763 |
| 5 | TI ( (Worklife OR “Work life” OR “Work-life” OR “Work-nonwork” OR “Work self” OR “Work-self”) AND (Balance OR Conflict OR Equilibrium OR Relation* OR integration OR harmony OR blend) ) OR AB ( (Worklife OR “Work life” OR “Work-life” OR “Work-nonwork” OR “Work self” OR “Work-self”) AND (Balance OR Conflict OR Equilibrium OR Relation* OR integration OR harmony OR blend) ) | 2151 |
| 6 | #4 OR #5 | 5122 |
| 7 | #3 AND #6 | 1378 |

((MH "Nurses+") OR (TI ( Nurs* OR “Enrolled nurs*” OR “Registered nurs*” OR “Community nurs*” OR “Acute nurs*” OR “Clinic nurs*” ) AND AB ( Nurs* OR “Enrolled nurs*” OR “Registered nurs*” OR “Community nurs*” OR “Acute nurs*” OR “Clinic nurs*” ))) AND ((MH "Work-Life Balance") OR (TI ( (Worklife OR “Work life” OR “Work-life” OR “Work-nonwork” OR “Work self” OR “Work-self”) AND (Balance OR Conflict OR Equilibrium OR Relation* OR integration OR harmony OR blend) ) OR AB ( (Worklife OR “Work life” OR “Work-life” OR “Work-nonwork” OR “Work self” OR “Work-self”) AND (Balance OR Conflict OR Equilibrium OR Relation* OR integration OR harmony OR blend) )))

**Supplementary Table 1 (continued)**

Database: PsycINFO (Ovid)

Date of Search: 04/04/2024

No. of Results: 369

Limits applied *(refer to PRISMA-S item 9)*: nil

Filters applied *(refer to PRISMA-S item 10)*: nil

| **S/N** | **Search terms** | **Number of results** |
| --- | --- | --- |
| 1 | exp Nurses/ | 39933 |
| 2 | (Nurs* or "Enrolled nurs*" or "Registered nurs*" or “Community nurs*” or “Acute nurs*” or "Clinic nurs*").ab,ti. | 118621 |
| 3 | #1 OR #2 | 119853 |
| 4 | exp Work-Life Balance/ | 1674 |
| 5 | (Worklife or "Work life" or "Work-life" or "Work-nonwork" or "Work self" or "Work-self").mp. and (Balance or Conflict or Equilibrium or Relation* or integration or harmony or blend).ab,ti. [mp=title, abstract, heading word, table of contents, key concepts, original title, tests & measures, mesh word] | 5364 |
| 6 | #4 OR #5 | 5561 |
| 7 | #3 AND #6 | 369 |

((exp Nurses/) OR ((Nurs* or "Enrolled nurs*" or "Registered nurs*" or “Community nurs*” or “Acute nurs*” or "Clinic nurs*").ab,ti.)) AND ((exp Work-Life Balance/) OR ((Worklife or "Work life" or "Work-life" or "Work-nonwork" or "Work self" or "Work-self").mp. and (Balance or Conflict or Equilibrium or Relation* or integration or harmony or blend).ab,ti. [mp=title, abstract, heading word, table of contents, key concepts, original title, tests & measures, mesh word]))

Database: Scopus

Date of Search: 04/04/2024

No. of Results: 1118

Limits applied *(refer to PRISMA-S item 9)*: nil

Filters applied *(refer to PRISMA-S item 10)*: nil

| **S/N** | **Search terms** | **Number of results** |
| --- | --- | --- |
| 1 | TITLE-ABS-KEY ( nurs* OR "enrolled nurs*" OR "registered nurs*" OR "community nurs*" OR "acute nurs*" OR "clinic nurs*" ) AND TITLE-ABS-KEY ( ( worklife OR "work life" OR "work-life" OR "work-nonwork" OR "work self" OR "work-self" ) AND ( balance OR conflict OR equilibrium OR relation* OR integration OR harmony OR blend ) ) | 1118 |

**Supplementary Table 1 (continued)**

Database: Web of Science

Date of Search: 04/04/2024

No. of Results: 615

Limits applied *(refer to PRISMA-S item 9)*: nil

Filters applied *(refer to PRISMA-S item 10)*: nil

| **S/N** | **Search terms** | **Number of results** |
| --- | --- | --- |
| 1 | ((TI=(Nurs* OR “Enrolled nurs*” OR “Registered nurs*” OR “Community nurs*” OR “Acute nurs*” OR “Clinic nurs*”)) OR AB=(Nurs* OR “Enrolled nurs*” OR “Registered nurs*” OR “Community nurs*” OR “Acute nurs*” OR “Clinic nurs*”)) AND ((TI=((Worklife OR “Work life” OR “Work-life” OR “Work-nonwork” OR “Work self” OR “Work-self”) AND (Balance OR Conflict OR Equilibrium OR Relation* OR integration OR harmony OR blend))) OR AB=((Worklife OR “Work life” OR “Work-life” OR “Work-nonwork” OR “Work self” OR “Work-self”) AND (Balance OR Conflict OR Equilibrium OR Relation* OR integration OR harmony OR blend))) | 615 |

**Appendices**

**Appendix A** Preferred Reporting Items for Systematic Reviews and Meta-Analyses 2020 Checklist (Page et al., 2021)

| **Section and Topic** | **Item #** | **Checklist item** | **Location where item is reported** |
| --- | --- | --- | --- |
| **TITLE** | | | |
| Title | 1 | Identify the report as a systematic review. | Page 1  Lines 2-3 |
| **ABSTRACT** | | | |
| Abstract | 2 | See the PRISMA 2020 for Abstracts checklist. | Refer to Appendix B |
| **INTRODUCTION** | | | |
| Rationale | 3 | Describe the rationale for the review in the context of existing knowledge. | Page 6  Lines 17-28 |
| Objectives | 4 | Provide an explicit statement of the objective(s) or question(s) the review addresses. | Page 6  Lines 28-31 |
| **METHODS** | | | |
| Eligibility criteria | 5 | Specify the inclusion and exclusion criteria for the review and how studies were grouped for the syntheses. | Page 7 line 24 - Page 8 line 8 |
| Information sources | 6 | Specify all databases, registers, websites, organisations, reference lists and other sources searched or consulted to identify studies. Specify the date when each source was last searched or consulted. | Page 7  Lines 17-22 |
| Search strategy | 7 | Present the full search strategies for all databases, registers and websites, including any filters and limits used. | Page 7  Lines 11-15 |
| Selection process | 8 | Specify the methods used to decide whether a study met the inclusion criteria of the review, including how many reviewers screened each record and each report retrieved, whether they worked independently, and if applicable, details of automation tools used in the process. | Page 9  Lines 2-11 |
| Data collection process | 9 | Specify the methods used to collect data from reports, including how many reviewers collected data from each report, whether they worked independently, any processes for obtaining or confirming data from study investigators, and if applicable, details of automation tools used in the process. | Page 9 line 24 - Page 10 line 2 |
| Data items | 10a | List and define all outcomes for which data were sought. Specify whether all results that were compatible with each outcome domain in each study were sought (e.g. for all measures, time points, analyses), and if not, the methods used to decide which results to collect. | Page 9  Lines 25-27 |
|  | 10b | List and define all other variables for which data were sought (e.g. participant and intervention characteristics, funding sources). Describe any assumptions made about any missing or unclear information. | Page 9  Lines 25-30 |
| Study risk of bias assessment | 11 | Specify the methods used to assess risk of bias in the included studies, including details of the tool(s) used, how many reviewers assessed each study and whether they worked independently, and if applicable, details of automation tools used in the process. | Page 9  Lines 12-22 |
| Effect measures | 12 | Specify for each outcome the effect measure(s) (e.g. risk ratio, mean difference) used in the synthesis or presentation of results. | N/A |
| Synthesis methods | 13a | Describe the processes used to decide which studies were eligible for each synthesis (e.g. tabulating the study intervention characteristics and comparing against the planned groups for each synthesis (item #5)). | N/A |
|  | 13b | Describe any methods required to prepare the data for presentation or synthesis, such as handling of missing summary statistics, or data conversions. | Page 10  Lines 4-15 |
|  | 13c | Describe any methods used to tabulate or visually display results of individual studies and syntheses. | N/A |
|  | 13d | Describe any methods used to synthesize results and provide a rationale for the choice(s). If meta-analysis was performed, describe the model(s), method(s) to identify the presence and extent of statistical heterogeneity, and software package(s) used. | N/A |
|  | 13e | Describe any methods used to explore possible causes of heterogeneity among study results (e.g. subgroup analysis, meta-regression). | N/A |
|  | 13f | Describe any sensitivity analyses conducted to assess robustness of the synthesized results. | N/A |
| Reporting bias assessment | 14 | Describe any methods used to assess risk of bias due to missing results in a synthesis (arising from reporting biases). | N/A |
| Certainty assessment | 15 | Describe any methods used to assess certainty (or confidence) in the body of evidence for an outcome. | N/A |

**Appendix A** (continued)

| **Section and Topic** | **Item #** | **Checklist item** | **Location where item is reported** |
| --- | --- | --- | --- |
| **RESULTS** | | | |
| Study selection | 16a | Describe the results of the search and selection process, from the number of records identified in the search to the number of studies included in the review, ideally using a flow diagram. | Page 11  Lines 3-8 |
|  | 16b | Cite studies that might appear to meet the inclusion criteria, but which were excluded, and explain why they were excluded. | N/A |
| Study characteristics | 17 | Cite each included study and present its characteristics. | Page 12-17 |
| Risk of bias in studies | 18 | Present assessments of risk of bias for each included study. | Page 12 – 17 |
| Results in individual studies | 19 | For all outcomes, present, for each study: (a) summary statistics for each group (where appropriate) and (b) an effect estimate and its precision (e.g. confidence/credible interval), ideally using structured tables or plots. | N/A |
| Results of syntheses | 20a | For each synthesis, briefly summarise the characteristics and risk of bias among contributing studies. | Page 11  Lines 10 – 12 |
|  | 20b | Present results of all statistical syntheses conducted. If meta-analysis was done, present for each the summary estimate and its precision (e.g. confidence/credible interval) and measures of statistical heterogeneity. If comparing groups, describe the direction of the effect. | N/A |
|  | 20c | Present results of all investigations of possible causes of heterogeneity among study results. | N/A |
|  | 20d | Present results of all sensitivity analyses conducted to assess the robustness of the synthesized results. | N/A |
| Reporting biases | 21 | Present assessments of risk of bias due to missing results (arising from reporting biases) for each synthesis assessed. | N/A |
| Certainty of evidence | 22 | Present assessments of certainty (or confidence) in the body of evidence for each outcome assessed. | N/A |
| **DISCUSSION** | | | |
| Discussion | 23a | Provide a general interpretation of the results in the context of other evidence. | Page 21 line 2 - page 24 line 23 |
|  | 23b | Discuss any limitations of the evidence included in the review. | Page 24 line 29 - page 25 line 3 |
|  | 23c | Discuss any limitations of the review processes used. | Page 25  Lines 3-7 |
|  | 23d | Discuss implications of the results for practice, policy, and future research. | Page 25 line 28 - page 26 line 23 |
| **OTHER INFROMATION** | | | |
| Registration and protocol | 24a | Provide registration information for the review, including register name and registration number, or state that the review was not registered. | Page 7  Lines 7-8 |
|  | 24b | Indicate where the review protocol can be accessed, or state that a protocol was not prepared. | Page 7  Lines 7-8 |
|  | 24c | Describe and explain any amendments to information provided at registration or in the protocol. | N/A |
| Support | 25 | Describe sources of financial or non-financial support for the review, and the role of the funders or sponsors in the review. | N/A |
| Competing interests | 26 | Declare any competing interests of review authors. | N/A |
| Availability of data, code and other materials | 27 | Report which of the following are publicly available and where they can be found: template data collection forms; data extracted from included studies; data used for all analyses; analytic code; any other materials used in the review. | Refer to supplementary files |

N/A = not applicable

**Appendix B** Preferred Reporting Items for Systematic Reviews and Meta-Analyses 2020 for Abstracts Checklist (Page et al., 2021)

| **Section and Topic** | **Item #** | **Checklist item** | **Location where item is reported** |
| --- | --- | --- | --- |
| **TITLE** | | | |
| Title | 1 | Identify the report as a systematic review. | Page 1  Lines 2-3 |
| **BACKGROUND** | | | |
| Objectives | 2 | Provide an explicit statement of the main objective(s) or question(s) the review addresses. | Page 3  Line 2 |
| **METHODS** | | | |
| Eligibility criteria | 3 | Specify the inclusion and exclusion criteria for the review. | Page 3  Line 10-11 |
| Information sources | 4 | Specify the information sources (e.g. databases, registers) used to identify studies and the date when each was last searched. | Page 3  Lines 7-8 |
| Risk of bias | 5 | Specify the methods used to assess risk of bias in the included studies. | Page 3  Lines 11-12 |
| Synthesis of results | 6 | Specify the methods used to present and synthesise results | Page 3  Lines 12-13 |
| **RESULTS** | | | |
| Included studies | 7 | Give the total number of included studies and participants and summarise relevant characteristics of studies. | Page 3  Line 14 |
| Synthesis of results | 8 | Present results for main outcomes, preferably indicating the number of included studies and participants for each. If a meta-analyses was done, report the summary estimate and confidence/credible interval. If comparing groups, indicate the direction of the effect (i.e. which group is favoured). | Page 3  Lines 14-20 |
| **DISCUSSION** | | | |
| Limitations of evidence | 9 | Provide a brief summary of the limitations of the evidence include in the review (e.g. study risk of bias, inconsistency and imprecision). | Page 3  Lines 25-27 |
| Interpretation | 10 | Provide a general interpretation of the results and important implications. | Page 3  Lines 21-25 |
| **OTHER** | | | |
| Funding | 11 | Specify the primary source of funding for the review. | Page 1  Lines 32 - 33 |
| Registration | 12 | Provide the register name and registration number. | N/A |

N/A = not applicable

**Appendix C** Peer Review of Electronic Search Strategies 2015 Evidence-Based-Checklist (McGowan et al., 2016)

| **Criteria** | **Tick** |
| --- | --- |
| **TRANSLATION OF THE RESEARCH QUESTION** | |
| Does the search strategy match the research question/PICO? | Yes |
| Are the search concepts clear? | Yes |
| Are there too many or too few PICO elements included? | No |
| Are the search concepts too narrow or too broad? | No |
| Does the search retrieve too many or too few records? | No |
| Are unconventional or complex strategies explained? | N/A |
| **BOOLEAN AND PROXIMITY OPERATORS** | |
| Are Boolean or proximity operators used correctly? | Yes |
| Is the use of nesting with brackets appropriate and effective for the search? | Yes |
| If NOT is used, is this likely to result in any unintended exclusions? | N/A |
| Is the width of proximity operators suitable (e.g., might adj5 pick up more variants than adj2)? | N/A |
| **SUBJECT HEADINGS (DATABASE-SPECIFIC)** | |
| Are the subject headings relevant? | Yes |
| Are any relevant subject headings missing; for example, previous index terms? | No |
| Are any subject headings too broad or too narrow? | No |
| Are subject headings exploded where necessary and vice versa? | N/A |
| Are major headings (“starring” or restrict to focus) used? If so, is there adequate justification? | N/A |
| Are subheadings missing? | N/A |
| Are subheadings attached to subject headings? | N/A |
| Are floating subheadings relevant and used appropriately? | N/A |
| Are both subject headings and terms in free text used for each concept? | Yes |
| **TEXT WORD SEARCHING (FREE TEXT)** | |
| Does the search include all spelling variants in free text? | Yes |
| Does the search include all synonyms or antonyms (e.g., opposites)? | Yes |
| Does the search capture relevant truncation (i.e., is truncation at the correct place)? | Yes |
| Is the truncation too broad or too narrow? | No |
| Are acronyms or abbreviations used appropriately? Do they capture irrelevant material? Are the full terms also included? | Yes |
| Are the keywords specific enough or too broad? Are too many or too few keywords used? Are stop words used? | No |
| Have the appropriate fields been searched; e.g., is the choice of the text word fields (.tw.) or all fields (.af.) appropriate? Are there any other fields to be included or excluded (database-specific)? | Yes |
| Should any long strings be broken into several shorter search statements? | Yes |
| **SPELLING, SYNTAX AND LINE NUMBERS** | |
| Are there any spelling errors? | No |
| Are there any errors in system syntax; e.g., the use of a truncation symbol from a different search interface? | No |
| Are there incorrect line combinations or orphan lines (i.e., lines that are not referred to in the final summation that could indicate an error in an AND or OR statement)? | No |
| **LIMITS AND FILTERS** | |
| Are all limits and filters used appropriately and are they relevant given the research question? | N/A |
| Are all limits and filters used appropriately and are they relevant for the database? | N/A |
| Are any potentially helpful limits or filters missing? Are the limits or filters too broad or too narrow? Can any limits or filters be added or taken away? | N/A |
| Are sources cited for the filters used? | N/A |

N/A = not applicable

**Appendix D** Mixed Methods Appraisal Tool version 2018 (Hong et al., 2018)

| **Category of study designs** | **Methodological quality criteria** | **Responses** | | | |
| --- | --- | --- | --- | --- | --- |
|  |  | Yes | No | Can’t tell | Comments |
| Screening questions (for all types) | S1. Are there clear research questions? |  |  |  |  |
|  | S2. Do the collected data allow to address the research questions? |  |  |  |  |
|  | Further appraisal may not be feasible or appropriate when the answer is ‘No’ or ‘Can’t tell’ to one or both screening questions. |  |  |  |  |
| 1. Qualitative | 1.1. Is the qualitative approach appropriate to answer the research question? |  |  |  |  |
|  | 1.2. Are the qualitative data collection methods adequate to address the research question? |  |  |  |  |
|  | 1.3. Are the findings adequately derived from the data? |  |  |  |  |
|  | 1.4. Is the interpretation of results sufficiently substantiated by data? |  |  |  |  |
|  | 1.5. Is there coherence between qualitative data sources, collection, analysis and interpretation? |  |  |  |  |
| 2. Quantitative randomized controlled trials | 2.1. Is randomization appropriately performed? |  |  |  |  |
|  | 2.2. Are the groups comparable at baseline? |  |  |  |  |
|  | 2.3. Are there complete outcome data? |  |  |  |  |
|  | 2.4. Are outcome assessors blinded to the intervention provided? |  |  |  |  |
|  | 2.5 Did the participants adhere to the assigned intervention? |  |  |  |  |
| 3. Quantitative non-randomized | 3.1. Are the participants representative of the target population? |  |  |  |  |
|  | 3.2. Are measurements appropriate regarding both the outcome and intervention (or exposure)? |  |  |  |  |
|  | 3.3. Are there complete outcome data? |  |  |  |  |
|  | 3.4. Are the confounders accounted for in the design and analysis? |  |  |  |  |
|  | 3.5. During the study period, is the intervention administered (or exposure occurred) as intended? |  |  |  |  |
| 4. Quantitative descriptive | 4.1. Is the sampling strategy relevant to address the research question? |  |  |  |  |
|  | 4.2. Is the sample representative of the target population? |  |  |  |  |
|  | 4.3. Are the measurements appropriate? |  |  |  |  |
|  | 4.4. Is the risk of nonresponse bias low? |  |  |  |  |
|  | 4.5. Is the statistical analysis appropriate to answer the research question? |  |  |  |  |
| 5. Mixed methods | 5.1. Is there an adequate rationale for using a mixed methods design to address the research question? |  |  |  |  |
|  | 5.2. Are the different components of the study effectively integrated to answer the research question? |  |  |  |  |
|  | 5.3. Are the outputs of the integration of qualitative and quantitative components adequately interpreted? |  |  |  |  |
|  | 5.4. Are divergences and inconsistencies between quantitative and qualitative results adequately addressed? |  |  |  |  |
|  | 5.5. Do the different components of the study adhere to the quality criteria of each tradition of the methods involved? |  |  |  |  |

**Appendix E** Customized data extraction checklist

Reviewer ___________________________	Date ___________________________

Title of article _______________________________________________________________

Author(s) of the publication ________________	Year _________	Country ____________

Type of study

| Quantitative study | □ |
| --- | --- |
| Qualitative study | □ |
| Mixed methods study | □ |

Study aim

Study design

Sample size and characteristics of participants

Data collection and outcome measurements

Key findings

**Appendix F** Quality appraisal of quantitative descriptive studies using the Mixed Methods Appraisal Tool version 2018 (Hong et al., 2018)

| **Author(s) (Year)** | **Article title** | **S1** | **S2** | **1.1-3.5** | **4.1** | **4.2** | **4.3** | **4.4** | **4.5** | **5.1-5.5** | **Score** | **Percentage** |
| --- | --- | --- | --- | --- | --- | --- | --- | --- | --- | --- | --- | --- |
| Cohen & Kirchmeyer (2005) | A cross-cultural study of the work/nonwork interface among Israeli nurses | Y | Y | N/A | Y | Y | Y | CT | Y | N/A | 6/7 | 85.71% |
| Michael Jeba Arasi & Sathish (2022) | A cross sectional survey on work life balance among Indian nurses during second wave of Covid–19 Pandemic | Y | Y | N/A | Y | Y | CT | CT | Y | N/A | 5/7 | 71.43% |
| Lee et al. (2020) | A longitudinal study of nurses' work-life balance: a case of a regional teaching hospital in Taiwan | Y | Y | N/A | Y | Y | Y | CT | Y | N/A | 6/7 | 85.71% |
| Dyrbye et al. (2020) | A national study of personal accomplishment, burnout, and satisfaction with work–life integration among advance practice nurses relative to other workers | Y | Y | N/A | Y | Y | Y | CT | Y | N/A | 6/7 | 85.71% |
| Navajas-Romero et al. (2020) | Analyzing the job demands-control-support model in work-life balance: a study among nurses in the European context | Y | Y | N/A | Y | Y | Y | CT | Y | N/A | 6/7 | 85.71% |
| Dyrbye et al. (2019) | Burnout and satisfaction with work–life integration among nurses | Y | Y | N/A | Y | Y | Y | Y | Y | N/A | 7/7 | 100% |
| Shiffer et al. (2018) | Effects of clockwise and counterclockwise job shift work rotation on sleep and work-life balance on hospital nurses | Y | Y | N/A | Y | Y | CT | Y | Y | N/A | 6/7 | 85.71% |
| Oh & Cho (2020) | Effects of nurses’ shiftwork characteristics and aspects of private life on work-life conflict | Y | Y | N/A | Y | Y | Y | CT | Y | N/A | 6/7 | 85.71% |
| Min (2022) | Effects of resilience, burnout, and work-related physical pain on work-life balance of registered nurses in South Korean nursing homes: A cross-sectional study | Y | Y | N/A | Y | Y | Y | CT | Y | N/A | 6/7 | 85.71% |
| Korkmaz Aslan et al. (2023) | Factors affecting work-life balance and psychological resilience levels of nurses working in internal clinics during COVID-19 | Y | Y | N/A | CT | CT | Y | CT | Y | N/A | 4/7 | 57.14% |
| Okayasu et al. (2022) | Factors associated with the work–self balance of nurses in an advanced medical center | Y | Y | N/A | Y | Y | Y | CT | Y | N/A | 6/7 | 85.71% |
| Sripo et al. (2019) | Factors related to work-life balance among occupational health nurses in Thailand | Y | Y | N/A | Y | Y | Y | CT | Y | N/A | 6/7 | 85.71% |

Y = yes, N = no, CT = can’t tell, N/A = not applicable

**Appendix F** (continued)

| **Author(s) (Year)** | **Article title** | **S1** | **S2** | **1.1-3.5** | **4.1** | **4.2** | **4.3** | **4.4** | **4.5** | **5.1-5.5** | **Score** | **Percentage** |
| --- | --- | --- | --- | --- | --- | --- | --- | --- | --- | --- | --- | --- |
| Ng et al. (2017) | Influence of job demands and job control on work-life balance among Taiwanese nurses | Y | Y | N/A | Y | Y | Y | Y | Y | N/A | 7/7 | 100% |
| Adiba & Nair (2021) | Occupational stress and its impact on work and work-life balance among nurses in a large private hospital in South India | N | CT | N/A | Y | Y | CT | CT | CT | N/A | 2/7 | 28.57% |
| Gribben & Semple (2021) | Prevalence and predictors of burnout and work-life balance within the hematology cancer nursing workforce | Y | Y | N/A | Y | Y | Y | Y | Y | N/A | 7/7 | 100% |
| Watanabe & Yamauchi (2016) | Psychosocial factors of overtime work in relation to work-nonwork balance: a multilevel structural equation modeling analysis of nurses working in hospitals | Y | Y | N/A | Y | N | CT | N | Y | N/A | 4/7 | 57.14% |
| Jena et al. (2021) | Sense of courage: The mediating role of courage between emotional reflexivity and work-life integration among nurses in Indian hospitals | Y | Y | N/A | Y | Y | Y | Y | Y | N/A | 7/7 | 100% |
| Rony et al. (2023) | The association between work-life imbalance, employees' unhappiness, work's impact on family, and family impacts on work among nurses: a cross-sectional Study | Y | Y | N/A | Y | Y | Y | CT | Y | N/A | 6/7 | 85.71% |
| Nurumal et al. (2017) | Work-life balance among teaching hospital nurses in Malaysia | Y | Y | N/A | Y | N | Y | CT | Y | N/A | 5/7 | 71.43% |
| Karunagaran et al. (2020) | Work-life balance of nurses during pandemic | Y | Y | N/A | Y | Y | Y | CT | Y | N/A | 6/7 | 85.71% |
| Terzi & Azizoğlu (2023) | Investigation of the relationship between workload perception and the work-life balance of intensive care nurses working during the COVID-19 pandemic: a web-based cross-sectional study | Y | Y | N/A | Y | Y | Y | CT | Y | N/A | 6/7 | 85.71% |

Y = yes, N = no, CT = can’t tell, N/A = not applicable

**Appendix G** Quality appraisal of quantitative randomized controlled trials using the Mixed Methods Appraisal Tool version 2018 (Hong et al., 2018)

| Author(s) (Year) | Article title | **S1** | **S2** | **1.1-1.5** | **2.1** | **2.2** | **2.3** | **2.4** | **2.5** | **3.1-5.5** | **Score** | **Percentage** |
| --- | --- | --- | --- | --- | --- | --- | --- | --- | --- | --- | --- | --- |
| Pryce et al. (2006) | Evaluation of an open-rota system in a Danish psychiatric hospital: a mechanism for improving job satisfaction and work–life balance | Y | Y | N/A | CT | CT | Y | CT | Y | N/A | 4/7 | 57.14% |

Y = yes, N = no, CT = can’t tell, N/A = not applicable

**Appendix H** Quality appraisal of qualitative studies using the Mixed Methods Appraisal Tool version 2018 (Hong et al., 2018)

| **Author(s) (Year)** | **Article title** | **S1** | **S2** | **1.1** | **1.2** | **1.3** | **1.4** | **1.5** | **2.1-5.5** | **Score** | **Percentage** |
| --- | --- | --- | --- | --- | --- | --- | --- | --- | --- | --- | --- |
| Dousin et al. (2021a) | The experience of work-life balance for women doctors and nurses in Malaysia | Y | Y | Y | Y | Y | Y | Y | N/A | 7/7 | 100% |

Y = yes, N = no, CT = can’t tell, N/A = not applicable
